# Integration of Newly Arrived Refugee Children into the German School System

**DOI:** 10.3390/ijerph18157854

**Published:** 2021-07-25

**Authors:** Pia Jäger, Notburga Ott, Angela Brand, Karim Fereidooni

**Affiliations:** 1 United Nations University—Maastricht Economic and Social Research Institute on Innovation and Technology (UNU-MERIT), Maastricht University, Boschstraat 24, 6211 AX Maastricht, The Netherlands; a.brand@maastrichtuniversity.nl; 2 Section for Social Policy and Social Economy, Faculty of Social Sciences, Ruhr-University Bochum, Universitätsstr. 150, 44801 Bochum, Germany; notburga.ott@rub.de; 3 Faculty of Social Sciences, Ruhr-University Bochum, Universitätsstr. 150, 44801 Bochum, Germany

**Keywords:** integration centers, school assignment, mental health, refugee children

## Abstract

*Background*: The assignment of newly arrived refugee children to the differentiated German school system represents a major challenge for the responsible municipalities. In this explorative research approach, the current assignment procedure, in addition to the necessary assessment of performance and the detection of learning, mental, or social disabilities of newly arrived refugee children in North Rhine-Westphalia (NRW), Germany, were investigated. *Methods*: Eight staff members of six relevant Communal Integration Centers (CICs) in NRW were interviewed and a qualitative content analysis was conducted. *Results*: The current assignment practices varied widely. The binding to guidelines differed; additionally, the school assignment or recommendation largely depended on personal engagement, connections, and attitudes of the relevant CIC staff. None of the CICs used standardized instruments. Instead, the staff assessed the performance with self-developed strategies such as free and playful approaches or self-developed worksheets, and counted on their ‘gut feeling’ and professional experience. *Conclusion*: The school career and education of newly arrived refugee children in NRW is largely inconsistent and dependent on the responsible CIC (e.g., the allocation of the family) and on the counseling staff member. Additionally, it must be assumed that relevant disabilities remain largely undetected.

## 1. Introduction

In 2016, more than 260,000 applications for asylum were submitted by children and adolescents aged 0 to 18 in Germany [1]. The majority of these applications were made by persons from Syria, Afghanistan, and Iraq. According to the Central Register of Foreigners, as at 28 February 2017, 423,577 children and adolescents from these countries lived in Germany.

### 1.1. Refugee Children as Lateral Entrants into the German Education System

For newly immigrated children with a refugee background, the framework conditions defined at the European level with regard to the beginning of compulsory education and the right to participate in the German education system apply. Member States are required to grant minors under international protection access to the educational system “on the same terms as their own nationals“ [2]. Therefore, access should be granted no later than three months after the submission of the application [3].

Due to their “cultural sovereignty“, the federal states are responsible for the organization of the school system according to the German Basic Law [4]. The assignment is primarily based on age and, if applicable, previous school certificates, or on the basis of official documents from the country of origin [5].

In North Rhine-Westphalia (NRW), the school assignments are usually made by the education authorities of the school ministry assigned to the district governments [6]. In NRW, 53 districts and independent cities have so-called ‘communal integration centers’ (CICs), which act as the contact point for the education counseling of the newcomers. For this purpose, the CICs conduct a detailed discussion on the initial consultation in which personal data, previous school careers, and educational experiences are recorded and knowledge is ascertained [7]. The specific experience of the teachers appointed to the CIC should enable an assessment of the children’s or adolescents’ performance potential in order to allow the most appropriate assignment to the respective school types or the associated “retention class” [8]. A guideline for standardized entry procedure does currently not exist. Studies evaluating this initial consultation and assessment of performance or the resulting implementation or applicability in practice were not available at the time of this research project.

Within the individual municipalities, different concepts are applied to these ‘newcomers’. An increasing number of municipalities offer so-called ‘language retention’ or ‘preparatory’ or ‘international specialization’ classes, which are offered parallel to or partially integrative with the regular lessons. In other municipalities, there is a direct assignment to the regular classes with accompanying language support [9,10]. The integration of the high number of newly immigrated children in the years 2015–2017 posed major challenges for many mainstream schools in the individual municipalities, particularly in NRW [11].

### 1.2. The Differentiated German School System

The introduction of compulsory education in the German school system during the 20th century was anchored in the meritocratic principle [12]. This made a decisive contribution to the education provided by the different types of schools that exist in Germany today. At the beginning of the 20th century, the selection of students in secondary schools (from grade four or six) was legitimized by the pursuit of homogeneous learning groups.

The primary level comprises the first four school years in primary school. In some federal states there is a six-year primary school or an orientation level in the fifth and sixth grades, independent of the type of school, which already counts as lower secondary level.

In Germany, the school system is highly differentiated with at least three, or even four, ‘basic’ types of school up to the secondary school level. This is predominantly based on the performance of the children and adolescents, and offers a variety of education graduations [13,14].

**Figure 1:**
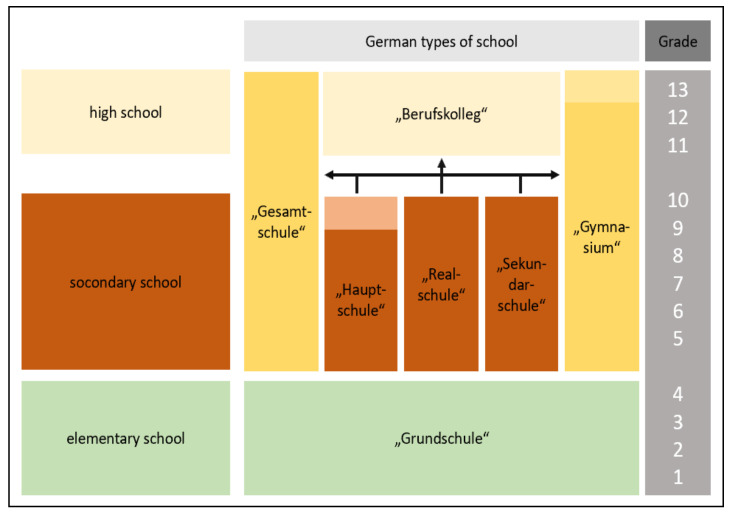
Overview of the German school system and its proper names (source: [15], with permission of the author).

An overview of the German school system, including its proper names, is presented in Figure 1, although it should be noted that some federal states differ from this general system due to specific regulations.

The lower secondary level leads to a grade that allows either the commencement of vocational training or transfer to a higher secondary level in a grammar school. The lower secondary level is divided into ‘Hauptschule’, ‘Realschule’, ‘Sekundarschule’, and ‘Gymnasium’, or integrated into a comprehensive school (in German: ‘Gesamtschule’). The higher secondary level begins after the lower secondary level as ‘gymnasiale Oberstufe’, which is comparable to ‘high school’ or ‘berufsbildende Schule’, including vocational training [16].

To improve readability for the international reader, the German proper names are translated as described in the Materials and Methods section. However, the distinction between the different types of school is retained. School assignment for newly arrived children is only necessary due to the absence of an inclusive school system in Germany and, therefore, is the topic of this article.

### 1.3. Research Desideratum Concerning the Inappropriate School Assignments

Due to the particularity of the non-inclusive school system in Germany, there is a lack of national and international research. This is true concerning both inappropriate school assignment in general, in addition to school assignment practice for newly arrived lateral entrants. Further evidence in this field of research is desirable.

### 1.4. Theoretical Perspective

#### 1.4.1. Children and Adolescents Being War Refugees

Both the causes of flight and the flight itself are often associated with the experience of traumatic events in children with refugee backgrounds [17].

This may—but does not necessarily—lead to or favor the appearance of mental disorder. Post-traumatic stress disorder (PTSD) is of particular relevance as a specific trauma disorder. Furthermore, psychosomatic, depressive, or anxiety disorders, in addition to a large number of other diseases, can occur as a result of the manifold burdens [18]. PTSD can present as both a primary and a secondary disorder [19].

#### 1.4.2. Mental Burden and School Performance

The school success of a child is closely linked to his or her cognitive performance and psychosocial development, which in turn can have a significant impact on a mental illness. For example, PTSD, which has the main symptoms of intrusions, avoidance behavior, and agitation (‘hyperarousel’), is often associated with impaired concentration. In children, it is often accompanied by a limitation of academic performance and school success [18,20]. For children and adolescents, depressive diseases are accompanied by impairment of concentration, social relationships, academic performance, and school achievement [21], in addition to limitations in cognitive performance [22].

The adequate diagnosis of and therapy for trauma-related disorders are therefore of particular relevance for the optimization of the prerequisites for successful integration into the German education system.

Each newly arrived refugee child undergoes not only an initial consultation with a CIC, but also an examination for school entry by the Health Office. This examination aims to detect both physical and mental disorders that may affect the school performance. However, previous research indicates that this goal cannot be achieved: there is an inappropriate under-detection of mental disorders and disabilities [23]. Thus, appropriate assessment of academic performance is extremely important. It is also equally important that an appropriate school assignment is provided that allows the newly arrived refugee children to receive the support they need.

#### 1.4.3. Children with Migrant Background in the German Education System

Various studies show that the factor of “migrant background” represents itself as a negative predictor for school success in the German education system, taking into account covariate influences [24,25].

In this regard, children with a refugee background are burdened by additional risk in their school development or integration: first, due to the risk of developmental limitations in school performance caused by mental stress or the disabilities associated with the experiences of flight; and, second, because of the disadvantages in the education system that already exist independently of the specific migration experiences due to the migrant background and the existing acculturation requirements.

### 1.5. Aims

The municipal and provincial systems responsible for the school recommendations and the assignments to the different types of schools are therefore faced with a particular challenge.

The aim of this investigation was to obtain the first scientific results regarding the school assignment and diagnosis of performance in newly immigrated children or young people with a refugee background through an exploratory qualitative survey, whereby the focus is on the work of the CIC. Resources and challenges, in addition to experiences with the implementation of educational diagnostics and recommendations in school integration, are explored.

## 2. Materials and Methods

### 2.1. Participants and Recruitment

By contacting CICs in NRW and the district government of Arnsberg, CIC staff members who were entrusted with the initial consultation of immigrant children and young people with a refugee background were identified and recruited as interview partners. A total of 10 staff members were contacted, eight of whom agreed to take part. One staff member did not respond to the request and, with another staff member, a suitable appointment time could not be found.

### 2.2. Informed Consent

The participants were informed in detail both in writing and verbally about the investigation and agreed to the audio recording of the interview, the qualitative analysis of the records, and the use of their information in anonymized form. A written informed consent form was signed by all of the interviewed CIC staff members.

### 2.3. Qualitative Assessment

This exploratory investigation was carried out by problem-centered interviews [26]. According to Witzel, the problem-centered interview is a theory-generating method based on both grounded theory and inductive-deductive approaches. It aims to focus on a specific topic from the subjects’ point of view and to add theoretical knowledge by ‘elastic concepts’.

The purpose is to “neutralize the alleged contradiction between being directed by theory or being open-minded so that the interplay of inductive and deductive thinking contributes to increasing the user’s knowledge” [26].

In the context of this investigation, the CIC staff members served as the subjects. The initial consultation with newly arrived refugee children was investigated primarily from their perspective. The structured interview guideline and initial categories that were developed during the pretest were used in a sense of “object orientation” [26]. The subsequent process of category building, transformation, and discussion was performed as a “process orientated” [26] procedure.

### 2.4. Evaluation Methods

According to Mayring, qualitative content analysis [27] is a coding evaluation method that allows one to work deductively and inductively by using a theory-guided category system or by working out categories from the data material.

The qualitative content analysis is carried out using the following sequence of steps:

1. Determination of the material
  Action-guiding question: Which material should be examined?
2. Analysis of the development situation
  Action-guiding question: How and under what conditions was the material manufactured?
3. Formal characterization of the material
  Action-guiding question: What form does the material to be evaluated have?
4. Determination of the direction of analysis
  Action-guiding question: Which research question is applied to the material?
5. Theoretical differentiation of the question
  Action-guiding question: Which theoretical concept is suitable to answer the research question?
6. Determination of the analytical technique
  Action-guiding question: Which analysis technique (summary, explication, structuring) is chosen?
7. Definition of the units of analysis
  Action-guiding question: Which analysis units play a role? (Coding unit, context unit, evaluation unit)
8. Carrying out the material analysis
  Action-guiding question: How can the material be evaluated based on criteria?
9. Summary of the results
  Action-guiding question: How can the results be summarized and the research question answered?
10. Application of the quality criteria
  Action-guiding question: Does the analysis correspond to the quality criteria?

### 2.5. Theoretic Sampling

The interview partners were selected using “theoretical sampling” [28], which uses the principle of maximally contrasting case selection. This specific procedure of the criteria-guided case selection does not intend to represent the sample because the criterion of representativeness is not provided for qualitative samples. Rather, what is sought is a “theoretical saturation” [29], “which records all characteristics in the dimensions theoretically considered relevant”.

### 2.6. Conducting the Interviews

The interviews were conducted and partially standardized using a guideline. Interviews used targeted ad-hoc questions [26,30] and further open-ended inquiries regarding aspects mentioned by the interviewees. An open-ended question about further important aspects at the end of the interview allowed an exploratory approach to be used. At the beginning of the interview, an open question asked for a short description of the professional activity.

The interviews took place personally in the premises of the CICs, with the help of a recording device (Olympus^®^). During the pretest, one interviewed staff member refused an audio recording; here, manual transcripts were used. Before interviewing, all interviewees agreed with the audio recording. 

### 2.7. Anonymization

Information that might allow identification of the relevant person was excluded. Regarding citation of the transcripts, information that included data on the person, or the exact location of the CIC staff or other named persons, was anonymized by blacking.

### 2.8. Pretest

The semi-structured interview guideline was piloted in four interviews with the target group in a pretest. This pretest served as an initial pilot ‘approach’ to explore the field of research and specify the problem-centered questions. The following main analysis was then adapted on the basis of the pretest. Both guidelines are available on request from the authors. To ensure a standardized research design and procedure of data collection, the interviews conducted during the pretest were not included in the following analysis, which is the main content of this article.

### 2.9. Language

Although this investigation concerns school assignment for both children and adolescents, in the following only the term “children” (meaning both children and adolescents) is used to improve readability.

Because the German school system has unique characteristics, the different types of schools cannot be translated easily.

To retain the sense of these school types and the aim of this investigation, which focused on the conditions and consequences regarding the assignments to the relevant school types, the different German school types are nonetheless differentiated. To improve readability for international readers, the German proper names of the school types that are described in the introduction are translated as follows:

“Hauptschule”—”basic modern secondary school”

‘Realschule’—’advanced modern secondary school’

‘Sekundarschule’—’comprehensive modern secondary school’ 

‘Gesamtschule’—’comprehensive school’

‘Gymnasium’—’grammar school’

‘Berufsschule’/‘Berufskolleg’—’vocational school’

Citations of statements were translated from German (in the following abbreviated as “TfG”). With the exception of minor linguistic smoothing, statements were maintained in their original form. Therefore, neither a grammatical nor a linguistic correction was carried out. Citations are referenced according to the Transcript (T) number and the respective Line (L).

## 3. Results

A total of eight employees of five different CICs in NRW were interviewed between 07/2017 and 07/2018. Three of the CICs were each responsible for an independent municipality. Two were allocated to a more rural area and therefore responsible for a circle, which means a special area containing several towns.

**Figure 2:**
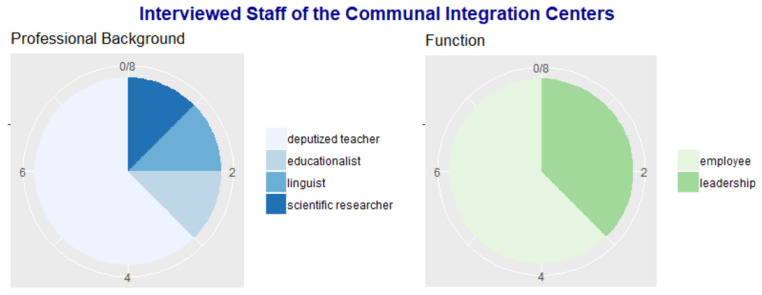
Information on the interviewed staff of the CICs.

All of the interviewed employees were female, having an age ranging from 32 to 50 years. The majority of the interviewed staff in the CICs (six of eight) worked previously as teachers and represented all of the major types of secondary schools. The professional background is presented in Figure 2.

Using the qualitative content analysis according to Mayring (see Materials and Methods), the categories that were developed deductively during the pretest were transformed based on an inductive procedure. In addition, subcategories were generated for several wide-ranging categories. 

**Figure 3:**
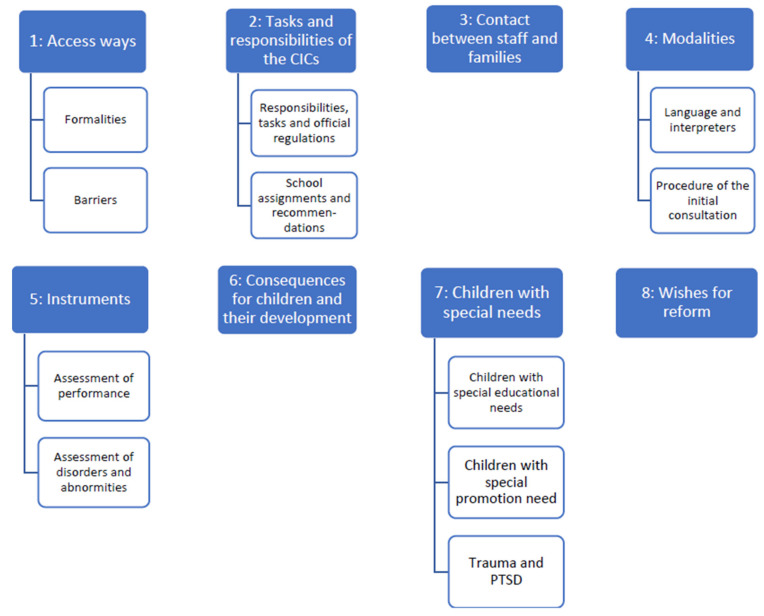
Category system developed using the qualitative content analysis.

As a result, a category system which is demonstrated in the following Figure 3 was developed.

### 3.1. Category 1: Access Ways

#### 3.1.1. Formalities

In the case of all of the included CICs, the newly arrived refugee families receive a letter from the responsible educational authority with a request to introduce themselves at the center. At the same time, the CICs receive the information about the newly arrived school-aged children. Therefore, they report feedback to the educational authority on the visit of the families.

In some municipalities, the newly arrived refugee families with school-aged children additionally received a flyer from the CIC during their registration in the municipal register.

Five of the eight interviewed persons reported that the target group is informed about having to introduce themselves at the CIC via diverse non-official means, such as word of mouth by other refugee families, voluntary workers, refugee workers, or their social network. This contact mostly takes place before the families receive the official postal request.

One of the interviewed staff members of a rurally located CIC reported that consultations also take place in the domestic environment if there is a poor traffic connection: “If there is no um public transport, no connection, then we go out […] and [um] then consult on the spot” (TfG, T. 5, L. 149—154).

#### 3.1.2. Barriers

Consistently, the interviewees reported that the process of contacting the school-aged children and their families was undertaken without any problems in the majority of cases. 

One reason given by the CIC staff related to difficulties in the understanding of the letter received from the education authority, which is written in German only. “There are people who do not want to know, to say the least” (TfG, T. 5, L. 149–154), said a CIC staff member. “Of course, these letters from the school office are always in German. That’s the official language, it also causes—I have the impression—a bit of anxiety in some families.” (TfG, T. 2, L. 160—187), another staff member said. “Two staff members of another CIC confirm this procedure: “often they [the refugee families] do not understand that letter” (TfG, T. 1, L. 274—276). None of the interviewed staff members reported offering invitation letters in another language than German.”

According to the interviewees, other reasons for difficulties during the contact process are ambiguities concerning formal information about the newly arrived families. Very rarely, there is a lack of cooperation by the parents.

If the parents of the school-aged children do not get in touch with the CIC, the education authority is engaged by the CIC staff. In these cases, delays may result. 

### 3.2. Category 2: Tasks and Responsibilities of the CICs

#### 3.2.1. Responsibilities, Tasks, and Official Regulations

The tasks and responsibilities vary widely between the single CICs. A common task is to collect data relating to the newly arrived refugee children. Additionally, the CICs connect schools and the new pupils, and explain the subsequent procedure to the parents. As an intermediate institution, the CICs are responsible for the school assignment of the newly arrived families, which includes organizing the contact with the assigned schools and the transmission of data to the educational authority. 

#### 3.2.2. School Assignments and Recommendations

**Figure 4:**
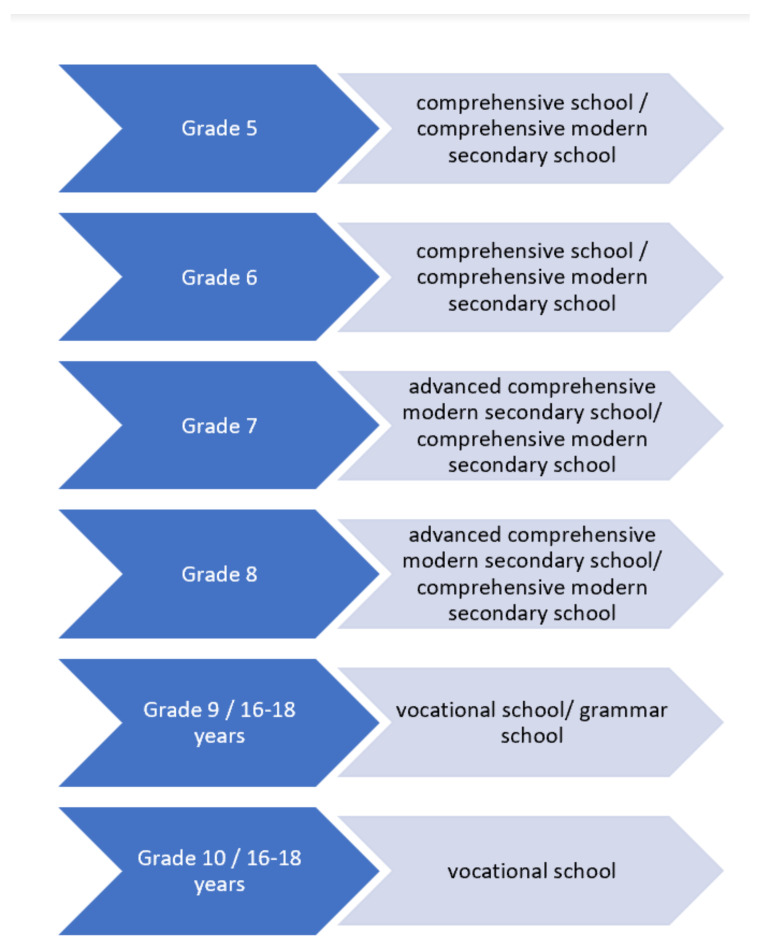
Guideline of the school inspectorate for the assignment of newly arrived refugee children in North Rhine-Westphalia.

The assignment procedure refers to both the assignment regarding the ‘type’ of school after elementary school and the grade. In the context of school assignments, all but one of the CICs carried out a personal consultation. The process of school assignment among these CICs was significantly different. In this analysis, three main procedures were identified:

1. Decree or guideline-based school assignments.
  Several CIC staff members reported that they were not able to make competency-oriented assignments up to 2015. Instead, after the decree of the educational office at the federal level came into force, the lower or upper school inspectorate developed guidelines for (or with) the educational authority. The assignments were based on the children’s grade to determine the types of school, as presented in Figure 4. In the case in which the guidelines differ for individual CICs, both possibilities are presented with a slash.
  Within the schools, there are special classes for language support with different names such as ‘welcome classes’ or ‘language remedial classes’ (see Introduction), which are parallel to the regular class. In most schools, no marks are awarded during the first two years, and therefore there is no pressure of having to repeat a class.
  The relevant CIC staff reported that this guideline is binding for all CICs and there is no scope of action for the individual CICs regarding the type of school. Therefore, they only have the opportunity to assign children to specific forms of school types, such as specializations of the ‘vocational schools’ or ‘grammar schools’ with specific language focuses. These aspects only play a role in big municipalities that offer a large number of different schools. Although one large municipality offers special classes for alphabetization, the other ones do not and proceed with these children as usual: “So illiterates are treated the same as everyone else. They must then also (.) grade nine ‘grammar school’ or grade seven ‘advanced modern secondary school’, or grade five or six ‘comprehensive school’” (TfG, T. 1; L. 965–967).
  Due to the absence of the influence on the school assignments, one of the questioned CIC stopped carrying out consultations. Instead, this CIC works only at an administrative level concerning the school assignment of refugee children. Therefore, the available data regarding the children from the offices are transferred to a form that is forwarded to the responsible school.
2. Exceptions while following, in general, the decree-based school assignments.
  Some CIC members reported following the guidelines of the school assignment in general but making exceptions for single ‘cases’. Most of the time, this unofficial procedure depends on the personal engagement and contacts of the respective CIC staff: “Sometimes I also call the headmaster and say: Maybe you would like to have a look at a student, I have one, who is great, just seven, I know, he would actually have to go to ‘advanced modern secondary school’, but check it out, please. Yes, and if I’m lucky, he does” (TfG, T. 1; L. 647–656).
3. Giving recommendations for competency-orientated assignments.
  In contrast, staff from other CICs reported on competency-oriented assignments and, therefore, wanting to assess the performance of the children. These CICs do not follow, or do not have to follow, a decree or a guideline. The staff members try to determine which type of school is the most adaptable and then give an appropriate recommendation. Although officially the educational authority is officially responsible for school assignments, the CIC staff gives recommendations and attempts to identify individual solutions for each child. In these CICs, the legal responsibility of school assignment also rests with the educational authority. However, the educational authority is not involved in the practical implementation of school assignments. As a result, the assignment is determined by the CIC staff member who carries out the consultation. Usually, the newly arrived refugee children and their families are only seen by one responsible CIC staff member. The type of school preferred by the responsible CIC staff largely depends on their previous experience. Five of the interviewed CIC staff members had been teachers before working at the CIC, and were experienced in all types of secondary schools in Germany. In addition, personal attitude plays a role. For example, one CIC employee reported that she prefers to assign children to less academic school types due to better support (TfG, T. 4; L. 87–106). This staff member had previously worked at a basic modern secondary school. In her experience, there is a better social and learning support at this ‘less academic’ type of school. As a result, she thinks that newly arrived refugee children are better suited to these schools, and prefers assigning children to this type of school. In contrast, another staff member had previously worked at a grammar school. She emphasized how difficult it is to catch up the academic level for children and adolescents who remain at less academic types of school after their arrival in Germany. Therefore, she is committed to assigning children who she thinks are suitable (see Section 3.5.2.) to grammar school.

In addition to the impression of the academic performance and the experience of the responsible CIC staff, ‘pragmatic’ aspects, such as residence proximity or having a social connection, are considered in the school assignment and regarding the grades (TfG, T. 5, L. 562–567).

As a result, the procedure of the school assignment is dependent on the municipality and the CIC to which the refugee family is allocated. Rather than a standardized procedure, the personal engagement and perspective of the responsible staff member determines the subsequent school career.

### 3.3. Category 3: Contact between Staff and Families

All of the seven CIC employees who make personal contact with newly arrived refugee families reported having good contact. They evaluated the contact as positive based on their experience of the consultations and conversations, which were usually friendly and kind. A staff member admits that “it cannot be generalized” (TfG, T. 1, L. 301) because it is “very different of course, […] very individual” (TfG, T. 7, L. 82–83). There is “a positive attitude” (TfG, T. 7, L. 84), “the contact is always very affectionate, very cordial” (TfG, T. 1, L. 304) with “families who are very open-minded across the board” (TfG, T. 5, L. 140–142). 

Inter alia, the staff members attribute this “positive contact” to the effort made by the CIC staff to generate a “pleasant atmosphere” (TfG, T. 7, L. 90) and “make the contact with them as pleasant as possible” (TfG, T. 5, L. 140). Thus, one of the interviewed staff members said “our aim is that every child laughs once in the consultation” (TfG, T. 1, L. 304–306). Therefore, the children to be counseled and their younger siblings were included in the consultation. There were possibilities for playing and painting. One CIC organized a “school welcome gift” based on donations.

Often, there are “questions beyond our actual topic” (TfG, T. 7, L. 90). One employee noted that “then they ask everything […] then such a school counseling quickly becomes such an all-encompassing consultancy” (TfG, T. 7, L. 234–247).

### 3.4. Category 4: Modalities

#### 3.4.1. Language and Interpreters

Because most of the families are newly arrived in Germany, “of course, the biggest challenge is very, very often the language” (T 5, L. 159–165). To make the initial consultation possible, three main strategies are used by the CICs to deal with this challenge.

1. Relatives, friends, or members of the “social network” of the refugee families who speak German and accompany the family to the consultations.
  A CIC staff member estimates “so right now, I would say: 90% of them are (um) come (um) directly with (um) any acquaintances, relatives, anyway” (TfG, T. 5, L. 866–879).
2. Those CICs that carry out initial consultations have a repertoire of several foreign languages spoken by their staff members.
  “So, the team is also good with the languages, so we are well-positioned, too” (TfG, T. 1, L. 384–385), an employee reported. Staff members of other CICs noted: “We speak here in the team Arabic, French, Greek, English, German of course (laughing) Polish, Russian, Turkish, Farsi […] that’s what we already cover” (TfG, T. 3, L. 174–177).
3. Volunteers who help with the translation.
  Three of the five CICs that give initial consultations reported having a pool of interpreters, language mediators or so-called “integration pilots”. Professional interpreters were not available in any of the CICs. “We could get some native speakers here to translate for us.” (TfG, T. 2, L. 191–223) or “have a linguistic mediator pool that we can use. […] These are mostly volunteers who are trained” (TfG, T. 5, L. 159–165 and L. 837–862), the staff reported.

Because the letter from the education authority is written in German, the necessity of an interpreter must be determined and organized during the appointment for the initial consultation. An employee responds to the question regarding whether the families know they should bring someone: “No, we do not make that public before, but that happens all by itself” (TfG, T. 7, L. 135–140).

If the need of an additional interpreter becomes obvious during the initial consultation, the meeting has to be carried out with significant language barriers or deferred in order to organize an interpreter. A staff member reports “then we do it with hands and feet (laughing) that somehow works, but [...] the education system, I cannot explain with my hands and feet” (TfG, T 7, L. 145–158).

#### 3.4.2. Procedure of the Initial Consultation

After the families have been contacted, all of the interviewed CICs that carry out consultations organize appointments. The families are asked to bring relevant documents and certificates. The CIC staff receive general information including personal data from the educational authority. Some of the CICs use additional questionnaires.

All of the seven CIC employees that were involved in initial consultations reported that the previous school career of the children and, in particular, the number of years of school attendance is included in experience.

Depending on the aspects that are decisive for the school assignment, further information is collected during the initial consultation with the families. Thus, the mentioned CIC that is located in a municipality and offers special “alphabetization classes” checks the literacy of the children. One CIC of a big municipality with several “grammar schools” with different language branches and bilingual education focuses on foreign language skills and previous foreign language teaching: “then foreign languages are always criteria, we have schools in XXX that offer English or French bilingually, this is also a criteria […] to find an assessment: Is this now a child, that is perhaps suitable for ‘grammar school’ later?” (T. 6, L. 145–153).

### 3.5. Category 5: Assessment Tools

#### 3.5.1. Instruments

With the exception of one of the interviewees, all of the CIC staff members denied using a standardized test, either for assessing the performance, or for relevant limitations, mental disabilities, social disorders, or learning disabilities. In addition to the language barrier, the reasons noted include the unavailability of suitable instruments and the specific situation during the initial consultation, which is not able to fully represent the performance and differences in the methods used within the countries of origin: “I believe that the children, when they arrive, are always in a special situation. […]. And when we do a test, (um), well, I do not know. I do not know how meaningful and capable it is” (TfG, T 6, L. 492–499), staff members reported. 

Additionally, time pressure and a high number of cases complicate standardized testing, “but XXX had so many numbers of cases that we rather worked it off just”, an employee reported (TfG, T 6, L. 682–636).

Standardized tests are only used in one CIC: an interviewee reported that, if the CIC staff recognizes a clear need for support, language-free tests are carried out with special educators who support the CIC team (TfG, T 4, L. 127–140).

#### 3.5.2. Assessment of Performance

To obtain an impression of skills and previous schooling, three main strategies of the CIC staff were identified:

1. Obtaining an impression due to a ‘free’ procedure.
  In this case, staff members use blank paper or oral tasks that help them to understand the level of achievement of the children.
2. Using self-developed worksheets.
  Three of the five CICs that carry out initial consultations reported on having developed some kind of ’test’ on their own: “We have developed our own consulting sheet here in the CIC, in the team” (TfG, T. 2, L. 189–190). The test mostly focuses on Maths and English, with the intention that these subjects depend less on German language skills. Two of these CICs use different materials depending on the grade of the children.
  One of the CICs only uses this test with children who are suspected of being unable to use the Latin alphabet: “in children, in which we think they cannot use the Latin alphabet, who are doing a test here?” (TfG, T. 6, L. 140–145).
3. School anamnesis, educational background of the family, and ‘gut feelings’.
  To obtain an overview of the previous school career, available certificates play an important role; however, a large number of refugee families do not have certificates. In addition, the school and education standards are hard to compare. An employee reported: “If they’ve been at a private school, then the children can speak perfect English [...] at a public school, then that is a little more difficult but top-performing children, you recognize them, too. […] I tell you, the gut feeling and the experience that you have over the years, which (...) has always been (knocking three times on the table) very well.” (TfG, T.3, L. 332–345).

In addition, the educational background and the engagement of the parents is important. An employee of another CIC reported: “If you ask the mother when her child went to school, or (um) last school year, what her child has visited, and then there appears a large question mark on (um) the mothers face and she says: I do not know. […]is it rather a mother, who is very much interested in, […] or is […]—where the probability is, um, that […] the child […] with the problem or with the quite everyday things, yes, well, has nobody who helps him” (TfG, T.5, L. 720–736).

Summarizing, the assessment of performance generated in the initial consultation relies on the staff’s ‘experience’ and ‘gut feeling’: “Interviewer: [...] so no standardized test at all? Staff member: No, No [...] That’s just the impression that this child gives me. Sometimes I have children sitting in front of me, they are burning and you realize that. They burn.” (TfG, T. 1; L. 666–673).

#### 3.5.3. Assessment of Disabilities and Disorders

Regarding both learning disabilities and mental or social disorders, the interviewed staff members reported that detection only takes place if parents bring corresponding documents, which means that a previous diagnosis must have taken place, mostly in the countries of origin: “I can only record these [meaning disorders] if the parents bring documents. Documents in the sense, either of the donating school, or there are psychological reports, reports by speech therapists” (TfG, T. 1; L. 783–794).

In contrast to physical disabilities, mental issues are often less obvious. However, the CIC staff encounter children and adolescents who have experienced significant mental traumas. Three of the CIC employees reported on having initial consultations with young girls who experienced sexual assault (one of which resulted in pregnancy) or forced prostitution.

The staff sometimes notice that there may be a mental disorder: “You have just this gut feeling, something is there, (.) there is, (.) is somehow strange” (TfG, T. 2; L. 600–602). Nevertheless, there is little possibility of detecting mental disorders: “Little to none […] we do not actually notice, somehow, that something is wrong” (TfG, T. 2; L. 802–808).

### 3.6. Category 6: Consequences for Individual Children and Their Development

Six of the interviewed CIC staff members assumed that an inadequate assignment to a school—either because a decree or guidelines must be followed, or due to restricted school places—may have a negative influence on the individual development of the children. 

Regarding the question of whether the interviewees think there is a disadvantage in educational success due to the guideline-based assignment, a staff member responded: “Definitely, definitely” (TfG, T. 1, L. 639–699). Another staff member was resigned: “I think so, but we have nothing else to offer. […] it comes to the point where you have to say: that’s it but what’s possible” (TfG, T. 4, L. 532–538). Some staff members assume that the subsequent school career will be decided after two years of a “language support class”: “So if I bring a child to ‘basic modern secondary school’, that does not mean it should stay there.[...] So the German system is very open, very flexible” (TfG, T. 3, L. 348–368).

A CIC staff member assumes that language acquisition is more significant than the school form. In addition, a school change may also have disadvantages. Several CIC members reported that they think the “comprehensive school” is an adequate type of school for nearly all children. One staff member regretted not having enough school places to allow assigning all children to the “comprehensive school”: “There they would have much more time to develop and to stay without marks a longer while, but I do not have the places.” (TfG, T. 6; L. 535–544) In other schools the capacities also are “tight, very tight” (TfG, T. 1; L. 757).

The opportunities of the newly arrived refugee children depend highly on the procedures and abilities of each municipality. However, the CIC staff members are not able to implement all of their preferred school assignments. In turn, the opportunities that are available influence the CIC staff in their consultation, in which they are able to use the resources as “effectively” as possible. Regarding the question of how recommendations for school forms can be implemented in practice, a CIC employee answered: “Sometimes more, sometimes less […] It becomes difficult then in the winter months, so where then really the school year has already started, and the classes are actually all relatively filled” (TfG, T. 7; L. 498–527).

Due to the non-detection of mental disorders, they often become obvious only on the long run: “If I think after two years, at the latest after two years: Here is nothing. By then, maybe I’ll get the hang of it: maybe something is not right here” (TfG, T. 4; L. 518–526).

### 3.7. Category 7: Children with Special Needs

#### 3.7.1. Children with Special Educational Needs

The CIC staff members noted different experiences of how families deal with their child’s disabilities: “We also have families, their children obviously have disabilities and they do not say it here because that’s something to be ashamed of” (TfG, T. 2; L. 938–942).

Depending on the municipality, special needs do not have an influence on the school assignment of children: “We now have no possibility here in this age group [meaning youths from 16–18 years] um to look for a special school due to for example a learning disability [...] ‘vocational schools’ are inclusive” (TfG, T. 7; L. 365–371). Sometimes, special needs become obvious during further attendance at school: “The child is coming to this ‘comprehensive modern secondary school’, just because that’s the only school that’s there. Um, then it goes in for a while and of course it might be that the (er) colleagues find out, or it is conspicuous for them, that something is wrong with the child” (TfG, T. 4; L. 277–298).’

#### 3.7.2. Children with Special Promotion Need

Once assigned to a school, it becomes difficult for refugee children to change to a “higher type” of school. In addition to this high barrier, staff members usually do not advise a school change to a “grammar school”. If the “comprehensive modern secondary school“ to which all refugee children were assigned “has a really gifted kid, and says, ok that’s it, we’ve had the kid for a year now. It is really outstanding, then they call me and we push the child practically to the next school but we really have a look on it. We also talk to the child and the family again and see if this really makes sense […] I can also go through at the “comprehensive modern secondary school“ until the end and then go to “grammar school“, then I have basically a normal way to school and do not have to go back and forth. Um, that’s why it’s really rare.” (TfG, T. 4; L. 51–61).

#### 3.7.3. Trauma and PTSD

Trauma-related disorders play an important role in the current schooling of refugee children. A staff member reported: “The biggest problem we have is the trauma […] that is getting more and more […] there is no indeed working help system for that” (TfG, T. 4; L. 445–458). As in the case of special educational needs, mental disorders become obvious over time. A CIC staff member estimated that the influence of PTSD on academic performance is “very high, because I think […] indeed the performance of those children will not rise, not get better and it is of course always on how they then [...] how they deal with it, the children and youths and how much support um possibilities they have of course” (TfG, T. 3; L. 521–530).

Regarding the treatment of trauma-related disorders, the CIC staff use personal connections: “and we have a (...) psychologist who came from Syria, worked there as a psychologist and offered to help here as well” (TfG, T. 1; L. 852–860). The CIC staff members assume that the teachers actually deal with trauma-related disorders the most: “yes, I mean our teachers are pretty fit in it right now” (TfG, T. 1; L. 852–855), an interviewee reported. Her colleague added: “there are also the school social workers at the schools” who support the teachers (TfG, T. 1; L. 855–860).

### 3.8. Category 8: Wishes for Reform

Regarding their own counseling setting, most of the staff members were satisfied: “here I am really happy with the way the handling is, how the structures are” (TfG, T. 5; L. 811–817). “At the moment I have the feeling that we have enough time for the individual consultations, […] that the families have enough space to ask questions” (TfG, T. 6; L. 509–521), a staff member of another CIC added.

Nevertheless, most of the staff members desired improvement regarding the recommendations and school assignments. The staff members that are bound to the guidelines desired a larger scope of action. From their perspective, the responsibility of the school assignments should be in the hands of the CIC that carries out the first consultation: “I would wish that, but unfortunately that is not wanted politically”, an employee reported.

Several of the questioned CIC staff members desired better support in dealing with mental issues, concerning both their own scope of action and the treatment possibilities in general. “So, I definitely need more professionals, for the special cases um, actually (.) these, these, these mental issues” (TfG, T. 4; L. 789–807).

To assign children appropriately, the CIC staff members demanded more school places (TfG, T. 2; L. 975–977). Additionally, the staff expressed the desire for better support for the parents of refugee children. One staff member said “I wish that the children and also the parents have help to be on their side, to help them with the first steps [...]the children know: which bus should I take now [...] and what do I have to do for it” (TfG, T. 1; L. 991–1007). In this context, the integration of newly arrived refugee families should be improved: “Yes, they are still pretty isolated in the leisure sector” (TfG, T. 1; L. 1079–1080).

## 4. Discussion

### 4.1. Consequences Regarding the Mental Health and School Success of Refugee Children

In this investigation, it was shown that, during the initial consultations, no detection of mental or social disorders or learning disabilities takes place. Although the staff members observe trauma-related disorders or learning disabilities, there is a lack of personal and professional competences to allow further steps to be taken. Several of the questioned CIC staff members desired better support in dealing with mental issues, concerning both their own scope of action and the treatment possibilities in general. Special needs have no influence on the school assignment of children:

Once assigned to a school, it is difficult for refugee children to change to a ‘higher type’ of school. In addition to this high barrier, the staff members usually do not advise a school change to a ‘grammar school’.

As in the case of special educational needs, mental disorders become obvious over time. Regarding the treatment of trauma-related disorders, the CIC staff members use their personal connections.

This observation is consistent with the current research results. Previous research indicates that the examinations of the health office for school entrants are not able to correctly detect mental disorders in refugee children [23]. Taking into account both the results of this analysis and those of previous research, it must be assumed that undetected relevant disorders persist in a large number of children. To create the best conditions for school success and personal development, the improvement of early detection in this high-risk group appear to be indispensable.

### 4.2. Inconsistent Procedure of School Assignment

The results of this *research* show that the practice of school assignment varies widely between the individual CICs. Three main procedures of school assignments were identified:

1. Decree or guideline-based school assignments.
  Several CIC staff members reported that they were not able to make competency-oriented assignments up to 2015. Instead, after the decree of the educational office at the federal level came into force, the lower or upper school inspectorate developed guidelines for (or with) the educational authority.
2. Making exceptions while generally following the decree-based school assignments.
  Some CIC members reported that they followed the guidelines for school assignment in general but making exceptions for single ‘cases’. Most of the time, this unofficial procedure depends on the personal engagement and contacts of the respective CIC staff member.
3. Giving recommendations to make competency-orientated assignments.
  In contrast, staff from other CICs report they make competency-oriented assignments and, therefore, want to assess the performance of the children. These CICs do not follow—or do not have to follow—a decree or a guideline. The staff members attempt to identify the type of school that is the most adaptable and then make an appropriate recommendation.

It was shown that, in some CICs, a guideline-based school assignment takes place but the staff make ‘exceptions’—which usually means an assignment to a ‘higher’ type of school—for single ‘cases’. The relevant staff members largely rely on their ‘gut feeling’ to detect gifted and ‘top-performing’ children. These ‘exceptions’ are possible due to the personal engagement and connections of the staff members.

Nevertheless, the question arises about whether students, who do not present themselves as ‘top-performing’ and have a family that are not as engaged, drop out or are disadvantaged due to this procedure. As a result, the subsequent school career of the newly arrived refugee children largely depends on the allocation of their families and, therefore, the responsible CIC, in addition to the ‘gut feeling’ and personal engagement of the counseling staff member.

### 4.3. Inconsistent Assessment of Performance and Previous School Education

Five of the eight interviewed persons reported that the target group is informed about having to introduce themselves at the CIC by a variety non-official means, such as word of mouth by other refugee families, voluntary workers, refugee workers, or their social network. This contact mostly takes place before the families receive the official postal request. One reason for this given by the CIC staff is the difficulty in understanding the letter received from the education authority, which is written in German only. According to the interviewees, other reasons for difficulties during the contact process are ambiguities concerning formal information about the newly arrived families. Very rarely, there is a lack of cooperation by the parents.

To make the initial consultation possible, three main strategies are used by the CICs to address this challenge:

1. Relatives, friends, or members of the ‘social network’ of the refugee families who speak German and accompany the family to the consultations.
2. The CICs that carry out initial consultations have a repertoire of several foreign languages that are spoken by their staff members.
3. Volunteers who help with the translation.

Each of the seven CIC employees who were involved in initial consultations reported that the previous school career of the children and, in particular, the number of years of school attendance, is relevant to their experience. Depending on the aspects that are decisive for the school assignment, further information is collected during the initial consultation with the families. Thus, the mentioned CIC, which is settled in a municipality that offers special ‘alphabetization classes’, assesses the literacy of the children.

With the exception of one of the interviewees, none of the interviewed CIC staff members used a standardized test, either to assess the performance, or the relevant limitations, mental disabilities, social disorders, or learning disabilities. In addition to the language barrier, the reasons cited included the unavailability of suitable instruments and the specific situation during the initial consultation, which was not able to fully represent the performance and differences in the methods used within the countries of origin.

To obtain an impression of skills and previous schooling, three main strategies of the CIC staff were identified:

1. Obtaining an impression due to a ‘free’ procedure.
  In this case, staff members use blank paper or oral tasks that help them to understand the level of achievement of the children.
2. Using self-developed worksheets.
  Three of the five CICs that carry out initial consultations reported that they developed some form of ‘test’ on their own.
  One of the CICs only uses this test with children who they suspect are not literate in Latin writing.
3. School anamnesis, educational background of the family, and ‘gut feelings’.
  To obtain an overview of the previous school career, available certificates play an important role. However, a large number of refugee families do not have certificates. In addition, it is difficult to compare school and education standards.

Interviewed staff members reported that detection of both learning disabilities and mental or social disorders only occurred if parents brought corresponding documents. This requires that a previous diagnosis must have taken place, which was most often in the countries of origin.

In contrast to physical disabilities, mental issues are often less obvious. However, the CIC staff encounter children and adolescents who have clearly experienced mental trauma. Three of the CIC employees reported that they had initial consultations with young girls who experienced sexual assault (one of which resulted in pregnancy) or forced prostitution.

Most of the interviewed CIC staff members assume that an inadequate assignment to a school—either due to a decree or guidelines that must be followed, or to a restricted number of school places—may have a negative influence on the individual development of the children.

The opportunities of the newly arrived refugee children highly depend on the procedure and ability of each municipality. However, CIC staff members are not able to implement all of their preferred school assignments. In turn, the opportunities that are available influence the CIC staff in their consultation, in which they are able to use the resources as ‘effective’ as possible. Although the staff members of the CICs who did not make competency-orientated assignments had reservations about making an assessment of performance, those who used competencies as much as possible to make their assignments attempted to use the initial consultations to obtain an impression of the academic performance. Different strategies of individual staff members could be developed to assess academic performance and previous school education, thus raising the question about the necessity of a standardized procedure.

Staff members spoke of individual ‘free’ procedures used to obtain an impression of each child. In contrast, several CICs developed their own grade-dependent ‘working sheet’, which means that, although they use an instrument for assessment, this instrument is only standardized within their own CIC. Furthermore, these instruments are not uniform, and an exchange of knowledge between the individual CICs does not take place. Most of these assessment procedures can be regarded as the result of the personal initiative of the CIC staff. Standardization and improvement of the relevant tools could allow consistent assessment and, in addition, provide relief to the employees.

### 4.4. Challenges and Disadvantages of a Guideline-Based School Assignment into Language Remedial Classes

In recent years, several approaches regarding the integration of newly arrived lateral entrants into the German school system have been developed. As presented in detail above, the current system mostly works with so-called language remedial courses that accompany regular classes.

Based on the experiences of the CIC staff, the guideline-based assignment offers opportunities but also presents disadvantages. Children often can, or must, change school after two years of language remedial classes. This provides opportunities for the development of the children; however, they may have to leave the ‘safe environment’ in which they have settled.

Additionally, moving into a ‘higher’ educational system may be difficult if there was an assignment to a lower educational system, even if the children have demonstrated their academic skills during the two years of language remedial class. The interviews indicated that the complications associated with the change in school increase with the age of the adolescents. In addition to the difficulties arising from missing certificates in grades that depend on certifications and legal gaps, the current practice of assigning youths from the age of 16 to ‘vocational schools’ therefore plays a crucial role. Although the ‘vocational school’ theoretically offers a range of certificates, it is difficult to obtain them in practice because all grades are obtained successively. A staff member determined that obtaining “Abitur” for a 17 or 18 year old student would take seven years, even if this degree was nearly finished in their home country. A high barrier exists for additional tests that may allow a marginal shortening of this time. Regarding the actual social and living situation of these young people, there is a wide range between the theoretical and actual opportunities within the German education system.

In contrast, the CICs that have a higher scope of action regarding the school assignments can possibly offer more opportunities to these youths. The personal engagement and connections offer educational opportunities that are not included in the guideline-based assignment procedure. Thus, these competency-orientated assignments enable a more individual procedure. This may also result in a dependency on the respective staff members, and the presented disadvantages or inconsistencies. 

Most staff members were satisfied with their own counseling setting. Nevertheless, most staff members desired to improve the recommendations and school assignments. The staff members who are bound to guidelines desired a larger scope of action. From their perspective, the responsibility of the school assignments should be with the CIC that carries out the first consultation.

To make an appropriate assignment, CIC staff members demand more school places. Additionally, the staff expressed a desire for better support for the parents of refugee children.

### 4.5. Limitations

This investigation was carried out with the best possible care and accuracy. However, the main limitations must be considered, both in terms of general limitations of a qualitative survey and the specific limitations of this investigation. The selection of the interview partners was cross-sectional, rather than randomized. In addition, we make no claim regarding representativeness. Our results demonstrate the procedure of school assignment in some municipalities and areas of NRW, and the associated problems. Due to the wide variance in the responsibilities of the CICs included in this survey, it may be that even greater deviations exist in other CICs that were not included.

## 5. Conclusions

In this explorative research, the current assignment procedure of newly arrived refugee children in NRW, Germany, was investigated. Therefore, eight interviews with staff members of six CICs in NRW were conducted and a qualitative content analysis was carried out. 

The access to and the contact between the employees, parents, and students occurred largely without or with few problems from the staff members’ perspective. The CICs developed different strategies to deal with language barriers and reported that these strategies were mostly effective. Most of the interviewed employees spoke of good contact without difficulties during the initial consultation. The three most important strategies for overcoming the language barriers are: the use of relatives, friends, or members of the “social network” of the refugee families who speak German and accompany them to the counseling sessions; the use of several foreign languages by their staff; and the use of volunteers who help with the translation.

However, the procedures varied significantly between these steps.

Almost all of the included CICs carried out personal initial consultations to assign the children and adolescents to the most suitable type of school, or to at least make a corresponding recommendation. Because the scope of action of the AI in the allocation practice is very different, three main procedures were determined: 1. School allocation, which is based on ordinances or guidelines; 2. Make exceptions to the guidelines, whereby the school allocation is generally based on valid ordinances; and 3. Make recommendations for making competence-based assignments.

The current assignment practice varies widely between the single CICs. The bonding to the assignment guidelines differs, and the school assignment or recommendation largely depends on personal engagement, connections, and attitudes of the relevant CIC staff. None of the CICs use standardized instruments, either to assess the performance or to detect mental or learning disabilities. Instead, the staff use self-developed strategies, such as free and playful approaches or worksheets, and relied on their ‘gut feeling’ and professional experience.

Although the area of influence on the school assignment is limited in some CICs, almost all employees carry out a small ‘performance diagnosis’ during initial consultations. During our analysis, three main assessment strategies were identified: make use of self-developed worksheets; establish the school career and educational background of the family; and personal ‘gut instinct’. Indeed, ‘gut instinct’ and personal preferences proved to be the most important aspects in referral practice. None of the employees use standardized tests to diagnose performance, either in assessing school performance or in determining intellectual, social, or learning disabilities. Two employees only focused on aspects that are actually relevant to their scope for flexibility in school assignments, namely, the level of literacy, foreign language skills, and the professional interest of young people up to the age of 16. Due to the lack of consequences of the initial counseling for school assignments, a CIC recently stopped providing initial counseling.

As a result, the subsequent school career and education of newly arrived refugee children in NRW is largely inconsistent and dependent on the responsible CIC, the allocation of the family, and the counseling staff member. Additionally, it must be assumed that relevant disabilities remain largely undetected.

The interviewed employees assume that the actual practice of school assignment may have a negative impact on subsequent school careers. Alternatively, the refugee children may benefit from the flexible German school system and a change from the assigned school type at a later point in time. Because impairments or disabilities are initially not recognized, teachers and employees of the CIC only become aware of them over time.

To cope with these special challenges, the employees of the CIC would like better professional support. Those CICs who are bound by a strict guideline with regard to school assignments would like more scope for flexibility.

Our results show that psychological or social impairments or learning disabilities are not recognized during the initial consultation. Although the staff encounter trauma-related disorders or learning disabilities in their daily professional lives, there is a lack of personal and professional skills to take further steps. Instead, these disorders only become visible over time, if the desired educational and developmental progress is not made or consequential damage occurs.

In all cases, an exchange between the CICs would be useful. In this context, the need for assessment tools and additional work materials should be discussed. In addition, tools that have been found to be helpful should be made available to all. A standardization and improvement of the respective work equipment could offer the possibility of a consistent assessment, and also provide relief to the CIC employees.

To generate more equal educational opportunities, a consistent procedure shared by all CICs and an exchange between the individual institutions would be desirable. Although a higher scope of action of the CICs regarding the school assignment could offer a more individualized procedure, this needs to provide the necessary conditions and instruments for the relevant CIC staff.

## Data Availability

All anonymized transcripts are available from the authors on request.

## Abbreviations

CIC	Communal integration centers
NRW	North Rhine-Westphalia
TfG	Translated from German
L	Line
T	Transcript
